# Medical Society Transactions in Journal Form

**Published:** 1903-10

**Authors:** 


### MEDICAL SOCIETY TRANSACTIONS IN JOURNAL FORM.

THE *Pennsylvania Medical Journal,* the official organ of the Medical Society of the State of Pennsylvania, was established in 1897, for the purpose of superseding the older method of publishing the transactions in an annual volume. Since that time, following its example, many State medical societies have adopted this means of recording their proceedings. Illinois, New York, Kansas, California, Michigan, Kentucky, Colorado, have adopted the system and other States are contemplating it. The advantages of this method of disseminating the official transactions over the annual bound volume plan are many, and recognition of these advantages is manifested by the fact that nearly every paper read before the society is also published in some journal as well as in the volume of transactions. No one wishes to have his paper buried from view, or, at most, be read only by the members of the society to whom the transactions are sent and who, in all likelihood, have heard the paper at the time it was read. Then why should a society incur the expense of issuing a bound volume when the papers could be vitalized in the columns of a journal, securing a wide audience and an attractive environment at less cost?

This will be a matter for the Georgia Medical Association to consider some day, and it is hoped that it will not have the epileptic aura that overtook it when the subject was broached some years ago.

